# Cloning and characterization of a pectin lyase gene from *Colletotrichum lindemuthianum *and comparative phylogenetic/structural analyses with genes from phytopathogenic and saprophytic/opportunistic microorganisms

**DOI:** 10.1186/1471-2180-11-260

**Published:** 2011-12-09

**Authors:** Alicia Lara-Márquez, María G Zavala-Páramo, Everardo López-Romero, Nancy Calderón-Cortés, Rodolfo López-Gómez, Ulises Conejo-Saucedo, Horacio Cano-Camacho

**Affiliations:** 1 Centro Multidisciplinario de Estudios en Biotecnología, Universidad Michoacana de San Nicolás de Hidalgo, Km. 9.5 Carretera Morelia-Zinapécuaro, Posta Veterinaria, Tarímbaro, C.P. 58000, Michoacán, México; 2 Departamento de Biología, División de Ciencias Naturales y Exactas, Universidad de Guanajuato, Apartado Postal No. 187, Guanajuato, Gto. 36000, México; 3 Instituto de Investigaciones Químico-Biológicas, Universidad Michoacana de San Nicolás de Hidalgo, Francisco J. Mujica S/N Col. Felicitas del Río, IIQB-Edif. B1, Morelia, Mich. 262, México

## Abstract

**Background:**

Microorganisms produce cell-wall-degrading enzymes as part of their strategies for plant invasion/nutrition. Among these, pectin lyases (PNLs) catalyze the depolymerization of esterified pectin by a β-elimination mechanism. PNLs are grouped together with pectate lyases (PL) in Family 1 of the polysaccharide lyases, as they share a conserved structure in a parallel β-helix. The best-characterized fungal pectin lyases are obtained from saprophytic/opportunistic fungi in the genera *Aspergillus *and *Penicillium *and from some pathogens such as *Colletotrichum gloeosporioides*.

The organism used in the present study, *Colletotrichum lindemuthianum*, is a phytopathogenic fungus that can be subdivided into different physiological races with different capacities to infect its host, *Phaseolus vulgaris*. These include the non-pathogenic and pathogenic strains known as races 0 and 1472, respectively.

**Results:**

Here we report the isolation and sequence analysis of the *Clpnl2 *gene, which encodes the pectin lyase 2 of *C. lindemuthianum*, and its expression in pathogenic and non-pathogenic races of *C. lindemuthianum *grown on different carbon sources. In addition, we performed a phylogenetic analysis of the deduced amino acid sequence of Clpnl2 based on reported sequences of PNLs from other sources and compared the three-dimensional structure of Clpnl2, as predicted by homology modeling, with those of other organisms. Both analyses revealed an early separation of bacterial pectin lyases from those found in fungi and oomycetes. Furthermore, two groups could be distinguished among the enzymes from fungi and oomycetes: one comprising enzymes from mostly saprophytic/opportunistic fungi and the other formed mainly by enzymes from pathogenic fungi and oomycetes. Clpnl2 was found in the latter group and was grouped together with the pectin lyase from *C. gloeosporioides*.

**Conclusions:**

The *Clpnl2 *gene of *C. lindemuthianum *shares the characteristic elements of genes coding for pectin lyases. A time-course analysis revealed significant differences between the two fungal races in terms of the expression of *Clpnl2 *encoding for pectin lyase 2. According to the results, pectin lyases from bacteria and fungi separated early during evolution. Likewise, the enzymes from fungi and oomycetes diverged in accordance with their differing lifestyles. It is possible that the diversity and nature of the assimilatory carbon substrates processed by these organisms played a determinant role in this phenomenon.

## Background

Pectin is one of the major components of the primary cell wall of plants and is also found in dividing cells and in the areas of contact between cells that have a secondary cell wall, including xylem and the fibrous cells of woody tissue. Pectin comprises approximately 35% of the primary cell wall of dicots and non-graminaceous monocots. Although its content in secondary walls is greatly reduced, it is believed that pectin plays an important role in the structure and function of both primary and secondary cell walls. The functions of pectin in cell walls are diverse and include plant growth and development, morphogenesis, defense, cell adhesion, cell wall structure, cellular expansion, porosity, ion binding, hydration of seeds, leaf abscission and fruit development, among others [1,2]. In general, pectin is considered to be a group of polysaccharides that are rich in galacturonic acid (GalA) and present in the form of covalently linked structural domains: homogalacturonan (HG), xylogalacturonan (XGA), rhamnogalacturonan I (RG-I) and rhamnogalacturonan II (RG-II) [1,2]. The main enzymes involved in the degradation of the HG backbone of pectin are polygalacturonases (PGA, E.C. 3.2.1.15 and XPG, E.C. 3.2.1.67), pectate lyases (PL, E.C. 4.2.2.9 and 4.2.2.2) and pectin lyases (PNL, E.C. 4.2.2.10) [3].

Pectin lyases (PNLs) catalyze the degradation of pectin through β-elimination; they remove a proton and generate an unsaturated bond between the C-4 and C-5 carbons of the non-reducing end of pectin, which is a neutral form of pectate in which the uronic acid moiety of galacturonic residues has been methyl-esterified. The activity of PNLs is highly dependent on the distribution of the methyl esters over the homogalacturonan backbone. PNLs exhibit pH optima in the range of 6.0-8.5 and, unlike PLs, their activity is independent of Ca^2+ ^ions; it is believed, however, that the residue Arg^236 ^plays a role similar to that of Ca^+2 ^[4,5]. Pectinase gene expression is regulated at the transcriptional level by the pH of the medium and by carbon sources, as it is induced by pectin and pectic components and repressed by glucose [6-8].

PNLs are grouped into Family 1 of the polysaccharide lyases [9] and into the pectate lyase superfamily that, in addition to pectin lyases and pectate lyases, also includes plant pollen/style proteins. The three-dimensional structures of five members of the pectate lyase superfamily have been determined. These include *Erwinia chrysanthemi *pectate lyase C (PELC) [10] and pectate lyase E (PELE) [11], *Bacillus subtilis *pectate lyase [12] and *Aspergillus niger *pectin lyase A (PLA) [13] and pectin lyase B (PLB) [14]. These enzymes fold into a parallel β-helix, which is a topology in which parallel β-strands are wound into a large right-handed coil [15]. Although PLs and PNLs exhibit a similar structural architecture and related catalysis mechanisms, they nonetheless diverge significantly in their carbohydrate binding strategy [4,13]. Currently, strategies are available for developing functional information from three-dimensional images of enzymes. The growing number of databases on the structure of pectinolytic enzymes has facilitated the analysis of minor structural differences that are responsible for the specific recognition of a unique oligosaccharide sequence in a heterogeneous mixture [4].

Most of the available information about fungal PNLs and their corresponding encoding genes has been obtained from saprophytic/opportunistic fungi such as *Aspergillus niger *[16-19], *A. orizae *[20,21], *A. fumigatus *[22], *Penicillium griseoroseum *[23], *P. occitanis *[24] and to a lesser extent from the phytopathogenic fungi *Glomerella cingulata *[25] and *C. gloeosporioides *[26].

The ascomycete *C. lindemuthianum *is an economically important phytopathogen, and along with its host *Phaseolus vulgaris*, it provides a convenient model to study the physiological and molecular bases of plant-pathogen interactions [27]. It is an intracellular hemibiotrophic pathogen with physiological races that invade the plant in an interaction consistent to the gene-for-gene model [28], and monogenic dominant resistance in common bean cultivars leads to the appearance of localized necrotic spots typical of the hypersensitive response (HR) [29]. After penetration of a host epidermal cell in a susceptible cultivar, the pathogenic races of *C. lindemuthianum *develop an infection vesicle and extend into adjacent cells by producing large primary hyphae, which invaginate without penetrating the host cell membrane and thus persist as a biotrophic interaction. Once a large area of the plant tissue has been colonized, necrotrophic hyphae develop [29], and this step closely correlates with the production of a number of host cell-wall-degrading enzymes that are characteristic of phytopathogenic fungi [30-32]. Up to know, race 0 is the only strain of *C. lindemuthianum *unable to infect *P. vulgaris*, which contrasts with 1472, one of the most virulent races isolated in México [33]. This difference makes the two races an excellent model to investigate the role of pectinolytic enzymes in virulence of *C. lindemuthianum*. Previous results from this laboratory revealed significant differences between pathogenic (1472) and non-pathogenic (0) races of *C. lindemuthianum *in terms of growth and production of extracellular PNL activity on different carbon and nitrogen sources in liquid culture. Accordingly, race 1472 grew faster in media containing glucose or polygalacturonic acid, and on 92%-esterified pectin, it produced levels of PNL activity that were approximately 2-fold higher than those produced by race 0. In contrast, cell walls isolated from *P. vulgaris *hypocotyls and, to a lesser degree, from cellulose sustained the growth of both races but induced PNL only in the pathogenic race [34].

Here we report the isolation and sequence analysis of the *Clpnl2 *gene, which encodes pectin lyase 2 of *C. lindemuthianum*, and its expression in pathogenic and non-pathogenic races of *C. lindemuthianum *in response to cultivation on different carbon sources. To determine the relationship among the three-dimensional structures of PNLs and the lifestyle of PNL-producing microorganisms, we performed a phylogenetic analysis using protein sequences and deduced amino acid sequences reported for PNLs. A comparative analysis of the three-dimensional structure of the Clpnl2 protein predicted by homology modeling, covering the main body of the protein and the carbohydrate binding site, and the three-dimensional structures of the PNLs used in the phylogenetic analysis was also performed.

## Methods

### Strain and culture conditions

*C. lindemuthianum *races 0 (non-pathogenic) and 1472 (pathogenic) were kindly provided by Dr. June Simpson (CINVESTAV-IPN, Unidad Irapuato, México) and maintained on potato dextrose agar (PDA, Difco) at 20°C. For DNA extraction, mycelia from *C. lindemuthianum *race 1472 grown on potato dextrose (PD) for 9 days at 20°C with continuous shaking (150 rpm), was recovered by filtration through Whatman paper No. 1 and stored at -85°C. For induction, 1.6 mg (about 5 cm^2^) of mycelia from races 0 and 1472 were inoculated in 250 ml-Erlenmeyer flasks containing 50 ml of PD medium and shaken (150 rpm) at 20°C. After 9 days, mycelia was collected by filtration, washed with water and transferred to 250 ml-Erlenmeyer flasks containing 50 ml of modified Mathur's medium (10 mM MgSO_4_.7H_2_O, 20 mM KH_2_PO_4_, 36 mM L-glutamic acid, distilled water up to 1 L; final pH, 5.5) [35] supplemented with either 2.5% glucose, 92%-esterified pectin or cell walls from *P. vulgaris*. Flasks were shaken (150 rpm) at 20°C and after different periods of growth, mycelia was collected by filtration, washed with water and stored at -85°C until use.

### Preparation of plant cell walls

Seedlings of *P. vulgaris *cv. Flor de Mayo were grown for 7 days, and cell walls were extracted and purified from hypocotyls as described elsewhere [36].

### DNA and RNA isolation

Genomic DNA was isolated from *C. lindemuthianum *mycelia that had been grown for 9 days in PD medium according to standard protocols [37]. Total RNA was purified from mycelia using TRIzol reagent (Invitrogen). RNA samples were treated with DNAse I according to manufacturer's instructions (Invitrogen) to eliminate DNA. The quality and concentration of total RNA were verified using the RNA 6000 Nano LabChip kit (2100 Agilent Bioanalyzer).

### Isolation of the homologous DNA *Clpnl2 *probe from *C. lindemuthianum*

Genomic DNA from race 1472 was amplified by PCR using the upstream primer pnlD (5'-CAGTACGTCTGGGGTGGTGA-3') and downstream primer pnlR (5'-AAGTAGTTGTTGACGACGTGG-3', which are homologous to sequences between 595 and 614 nt and 891 and 911 nt, respectively, of exon 3 of the *Clpnl2 *gene from *C. gloeosporioides *[GenBank: AAD43565]. The PCR incubation mixture was heated at 95°C for 5 min in a thermocycler (Eppendorf Master Cycler Gradient, Brinkmann, Westbury, NY), followed by denaturation for 1 min at 95°C, annealing for 2 min at 48°C and extension for 2 min at 72°C. PCR was then performed for 35 cycles, followed by a final extension for 10 min at 72°C. A PCR product of 383 bp corresponding to *pnl2 *gene (*clpnl2 *fragment) was ligated into the pCR 2.1 vector and introduced into *E. coli *TOP 10 strain from the TOPO TA Cloning kit (Invitrogen).

### Genomic DNA library construction and screening

Partial *Sau*3AI digestion of genomic DNA from race 1472 was used to construct a genomic library in Lambda DASH II/*Bam*HI according to manufacturer's instructions (Stratagene). Screening was performed using 15 × 10^4 ^UFP with three rounds of hybridization filters and the homologous *Clpnl2 *fragment, which was ^32^P-radiolabeled using the Radprime DNA Labeling System Life Technologies Kit (Tech-Line).

### Molecular cloning of the *Clpnl2 *full-length cDNA and expression analyses

The cDNA was amplified by RT-PCR as specified by the manufacturer. SuperScript III First-Strand Synthesis System for RT-PCR (Invitrogen) was used to prepare cDNA from total RNA. PCR was performed using the upstream primer Pnl67 (5'-ATGAAGTCTACCATCTTCTCCG-3') and downstream primer Pnl1569 (5'-TTAGATCTTGCGAAACCGGC-3') designed from the DNA *Clpnl2 *genomic sequence of *C. lindemuthianum*. The PCR incubation mixture was heated at 94°C for 5 min in a thermocycler (Eppendorf Master Cycler Gradient, Brinkmann, Westbury, NY), followed by 30 cycles of denaturation for 20 sec at 94°C, annealing for 30 sec at 54°C, extension for 1.5 min at 72°C and then by a final extension for 7 min at 72°C. A PCR product of 1,140 bp obtained from total RNA of race 1472 induced with pectin for 4 h and corresponding to the *Clpnl2 *gene, was ligated into the pCR 2.1 vector (Invitrogen) and three clones were selected and sequenced. The 5' end of cDNA was amplified by 5'RACE as specified by the manufacturer (5'RACE System for Rapid Amplification of cDNA Ends, Invitrogen), with total RNA from race 1472 induced for 4 h with 92%-esterified pectin, using the specific reverse primers Pnl1249 (5'-GTA GTT GTT GAC GAC GTG GAC G-3') and Pnl975 (5'-CGA TGT GCT GGC GGC CG-3'). The amplification products were cloned and five clones were selected and sequenced. For expression analysis, total cDNA (1140 pb) was amplified with specific primers Pnl67 and Pnl1569 in the same conditions described above using total RNA of mycelia from both races induced with 92%-esterified pectin or cell walls from *P. vulgaris *for 2, 4, 6, 8, 10 and 12 h. For expression analysis, cDNA obtained from cells grown under different conditions was also amplified by PCR using oligonucleotides prepared from ribosomal 18S RNA as a control (5'- TTAGCATGGAATAATRRAATAGGA-3'and 5'-ATTGCAATGCYCTATCCCCA-3) [38]. The PCR incubation mixture was heated at 94°C for 3 min, followed by 35 cycles of denaturation for 1 min at 94°C, annealing for 1 min at 56°C, extension for 1 min at 72°C and then a final extension for 10 min at 72°C. The cDNA of *Clpnl2 *and the amplified RT-PCR products were analyzed in a Bioanalyzer using the system for quantification and molecular size Agilent DNA 7500 (2100 Agilent Bioanalyzer).

### Southern blot hybridization

Genomic DNA of mycelia from race 1472 was digested with selected restriction endonucleases. Digestion products were size-fractionated on a 0.8% agarose gel, transferred to a nylon membrane (Hybond-N+, Amersham Pharmacia Biotec, England), hybridized and detected with a ^32^P-radiolabeled *Clpnl2 *probe. Hybridizations were carried out at 60°C in 2X SSC containing 0.5% blocking agent (Roche) and 0.1% SDS. After hybridization, the blot was washed at 60°C for 15 min with 2X SSC containing 1% SDS and then at 60°C for 15 min with 0.2X SSC containing 0.1% SDS.

### Sequencing and DNA analysis

The sequences of both strands of DNA of race 1472 and cDNA of both races were determined by the dideoxy-chain termination method using the ABI Prism Dye Cycle Sequencing Ready Reaction Kit in an ABI PRISM 310 DNA sequencer (Applied Biosystems, Foster City, CA). The nucleotide sequences were analyzed using the DNAsis (Hitachi) and 4Peaks v 1.7.2 software (http://mekentosj.com). *In silico *analyses of putative transcription factor binding sites were performed using the AliBaba2.1 software [39] and the Transfac 7.0 database [40]; the regulatory sequences reported for genes of fungal lytic enzymes were also compared. The N-terminal secretion signal sequence was identified with the SignalP 3.0 web server [41]. The protein molecular mass, pI and *N*-glycosylation sites were calculated on an ExPASy Proteomics Server [42].

### Phylogenetic analyses

Phylogenetic analyses were performed on the *Clpnl2 *deduced amino acid sequence and the deduced amino acid sequences of 34 pectin lyases that were previously reported (Table 1). Protein sequences were aligned with Clustal × software [43] using default parameters. Prior to phylogenetic analyses, signal peptide sequences and N-terminal and C-terminal extensions were excluded. Phylogenetic analyses were performed under Bayesian, maximum parsimony and neighbor-joining criteria, using the programs MrBayes Vs. 3.1.2 [44], PAUP*v 4b10 [45] and Mega 4 [46]. We used the amino BLOSUM G2 evolution model with gamma correction for Bayesian analysis. In total, 10,000 trees were obtained based on the settings ngen = 1000 000 and sample freq = 100 for Bayesian criteria. Prior to estimating the support of the topologies that were found, we checked the convergence of overall chains (4) when the log likelihood values reached the stationary distribution. The first 2500 trees were 'burn-in' and discarded, and a 50% majority rule consensus tree of the remaining trees was generated. For maximum parsimony analyses, the most parsimonious trees were estimated using the heuristic search option (TBR branch swapping, saving only a single tree in each case) with random sequence addition (five random replicates). Support was evaluated by bootstrap analysis using the full heuristic search option with 1000 replicates. For the neighbor-joining method [47], a JTT matrix was used, and 1000 bootstrap replicates were performed. We used the *A. thaliana *pectate lyase [GenBank: CAB41092] as an outgroup for pectin lyase analyses.

**Table 1:** Nucleotide and protein sequences of reported pectin lyases used for phylogenetic analyses.

Microorganism	Access number
*Aspergillus niger*	GenBank: CAD34589, GenBank: AAW03313, GenBank: CAA39305, GenBank: CAA01023, GenBank: ACE00421, GenBank: AAA32701
*Aspergillus nidulans*	GenBank: ABF50854
*Aspergillus oryzae*	GenBank: BAB82468, GenBank: BAB82467
*Aspergillus fumigatus*	Swiss-Prot: BOYCL3, Swiss-Prot: Q4WV10, GenBank: EAL91586, Swiss-Prot: Q4W156
*Aspergillus terreus*	GenBank: EAU31855, GenBank: EAU37973
*Aspergillus clavatus*	GenBank: EAW12911
*Emericella nidulans*	Swiss-Prot: Q5BA61
*Colletotrichum gloeosporioides*	GenBank: AAA21817, GenBank: AAD43565, GenBank: AAF22244
*Penicillium occitanis*	GenBank: ABH03046
*Penicillium griseoroseum*	GenBank: AF502280
*Neosartorya fischeri*	GenBank: EAW17753, Swiss-Prot: A1CYC2
*Pyrenophora tritici-repentis*	GenBank: XP_001934252, GenBank: XP_001930850
*Ustilago maydis*	GenBank: EAK86184
*Verticillium albo-atrum*	GenBank: XP_003001443
*Phytophthora infestans*	GenBank: XP_002909420, GenBank: XP_002903922
*Bacillus subtilis*	GenBank: BAA12119, GenBank: AAB84422
*Pectobacterium atrosepticum*	GenBank: CAG74408
*Pectobacterium carotovorum*	GenBank: AAA24856

### Protein homology modeling

The tertiary structure of the deduced amino acid sequence of *Clpnl2 *was predicted by homology modeling using the Swiss-Model Server [48] using Pel B from *A. niger *(PDB: 1qcxA) as template [14]. The prediction of three-dimensional structures of the deduced amino acid sequences used in the phylogenetic analysis was performed in a similar manner. The structural parameters and prediction quality of the modeled structures were evaluated using the program SPDBV v. 4.01 [49]. The energy minimization of the model was performed by GROMOS96 [50], which was provided by the SPDBV program. MMV 2010.2.0.0 (Molegro ApS) and SPDBV v. 4.01 were used for visualization of molecular structures.

### Multiple comparisons of protein structures

The comparison of protein structures was performed using the Voronoi contact method [51] with the ProCKSI-Server [52]. Calculations were performed using default parameters, and the resultant similarity matrixes (Voronoi-contacts) were standardized and used as the input for clustering of the protein set using the un-weighted pair group method for the arithmetic mean (UPGMA) [53].

## Results and discussion

### Isolation and sequence analysis of the *Clpnl2 *gene

Nine positive clones were isolated from the screening of a *C. lindemuthianum *genomic library using the ^32^P-radiolabeled fragment of *Clpnl2*. Southern blot analysis of the clones allowed the identification of a 4.0-kb fragment that hybridized with the PCR probe. The 4.0-kb fragment was subcloned, and 2,159 bp containing the *Clpnl2 *gene was sequenced [GenBank: JN034038]. The full-length *Clpnl2 *cDNA of races 1472 [GenBank: JN034039] and 0 [GenBank: JN653459] obtained by RT-PCR from total RNA of mycelium induced for 4 h with 92%-esterified pectin, was sequenced using specific primers designed from the *Clpnl2 *gene sequence.

**Figure 1:**
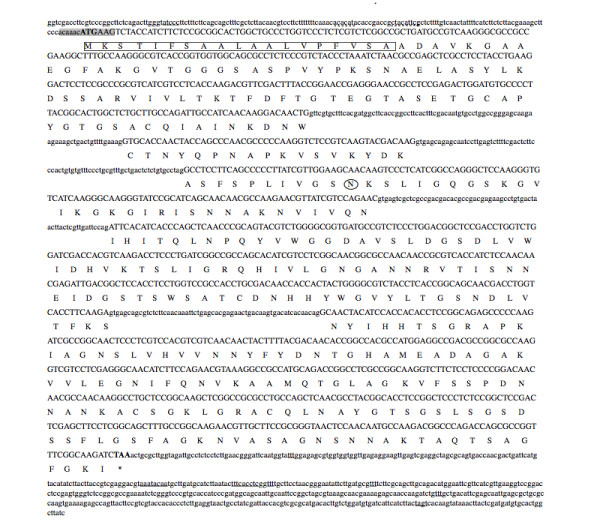
**Nucleotide and deduced amino acid sequence of the *Clpnl2 *gene**. Intron and exon sequences are in lowercase and uppercase, respectively. The signal peptide sequence is boxed. The possible binding sequences of RAP1 and AbaA are underlined with a dotted line. The putative transcription start point is underlined, and the putative Kozak sequence is shaded. The sequences of the 3'-terminal region are underlined. An asterisk (*) marks the translation stop codon. The potential N-glycosylation site is circled. This sequence has been deposited in the GenBank nucleotide sequence database under accession number JN034038.

The analysis of the cDNA sequences showed no differences between the two races. The coding region of the *Clpnl2 *gene consisted of 1428 bp interrupted by four introns ranging in size from 60 to 87 bp (Figure 1). According to the 5'RACE analysis, a putative transcription starting point was localized [19], and the context of the start codon ATG matched with the Kozak sequence for filamentous fungi [54]. Two possible regulatory sequences were identified in the 5' untranslated region of *Clpnl2*: a putative regulatory sequence for binding to RAP1, which is a transcriptional factor that participates in the activation of transcription and the silencing of genes in yeast cells, located at position +54 [55] and a possible binding sequence for the transcription factor AbaA at position +69. AbaA binding sites have been observed in several genes that participate in the control of cell development in organisms such as *A. nidulans *and the dimorphic fungus *P. marneffei*, where AbaA has been related to morphogenesis and dimorphism, respectively [56,57]. These putative regulatory elements were localized downstream the transcription site which is an uncommon finding. Multiple binding sites to AbaA have been reported in *cis *regulatory regions and some downstream the transcription starting site in *A. nidulans *genes. No attempts were made in this study to determine the function of these elements. Due to the size of the promoter region of *Clpnl2*, it was not possible to locate more elements commonly found in genes encoding for pectinolytic enzymes. The 5' and 3' untranslated regions (5'UTR and 3'UTR) were 129 and 563 bp, respectively. Two consensus sequences (AATAAA and TTTCACTGC) found in the terminal regions of eukaryotic mRNAs [58], and two of the three consensus sequences for yeast 3'-terminal regions (TAGT and YIT) [59] were detected in the *Clpnl2 *3'UTR.

The *Clpnl2 *cDNA contains an ORF of 1140 nucleotides that encodes a putative protein of 379 aa with a N-terminal secretion signal sequence of 19 amino acids, according to the SignalP 3.0 web server [41]. A protein of molecular mass 37.4 kDa and a pI of 9.1 was calculated, and one potential *N*-glycosylation site was located at position 110 (ExPASy Proteomics Server) [42]. These results are consistent with those reported for the amino acid sequences of *Pnl2 *and *pnlA *of *C. gloeosporioides *[25,26]. Despite *N*-glycosylation is common in pectinolytic enzymes and has been reported in several fungal pectin lyases al similar positions, little is known about the function of this posttranslational modification. Although it is believed that it affect enzyme stability and activity [60,61].

### Southern blot analysis

**Figure 2:**
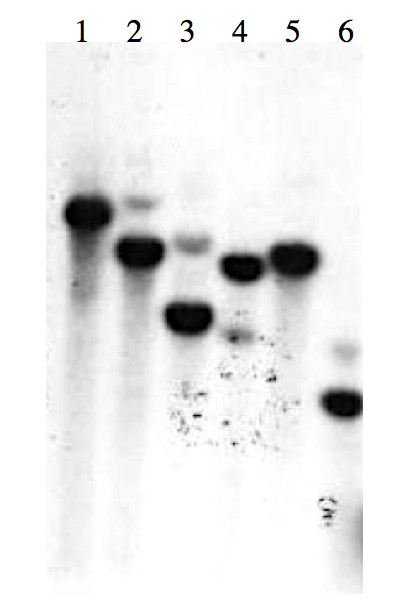
**Southern blot analysis of total DNA from *C. lindemuthianum***. Total DNA was digested with BamHI (1), EcoRI (2), HindIII (3), XhoI (4), EcoRI/BamHI (5), or HindIII/XhoI (6), analyzed on a 0.8% agarose gel, transferred to nylon membrane and hybridized with a ^32^P-radiolabeled *Clpnl2 *fragment.

The genomic organization of the *Clpnl2 *gene was investigated by Southern blot analysis. Total DNA was digested with the restriction endonucleases *Bam*HI, *Eco*RI, *Hind *III, *Xho*I, *Eco*RI/*Bam*HI and *Hind *III/*Xho*I. The digested DNA was fractionated on a 0.8% agarose gel and hybridized to the ^32^P-radiolabeled *Clpnl2 *probe. As depicted in Figure 2, commonly a single hybridization product was detected. In addition, a very faint signal probably resulting from hybridization with another gene of low similarity was observed. These results suggest that the *C. lindemuthianum *genome contains a single copy of the *Clpnl2 *gene, as does *C. gloeosporioides *[26].

### Protein homology modeling

**Figure 3:**
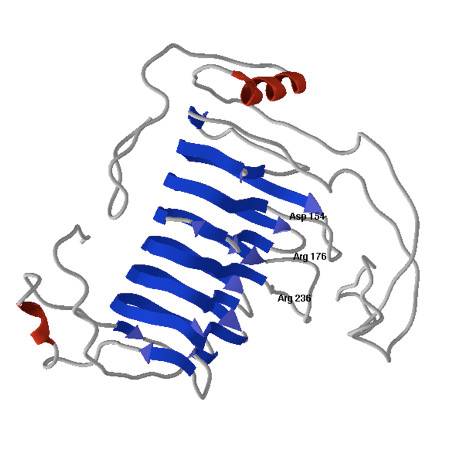
**Three-dimensional structure of *Clpnl2 *from *C. lindemuthianum *showing highly conserved residues involved in catalysis**.

The tertiary structure of *Clpnl2 *predicted by homology modeling coincided with the typical topology of the parallel β-helix of PNLs (Figure 3). After energy minimization, the energy value was -17418.428 kJ/mol, and the quality of the model generated was assessed by plating dihedrals Φ and Ψ onto Ramachandran plots (SPDBV v. 4.01) [49]. The results are in agreement with the requirements for preferred and allowed regions, except for 3 non-glycine residues (0.8%).

### Phylogenetic analyses

**Figure 4:**
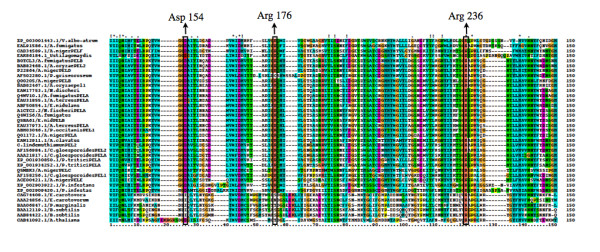
**Alignment of the amino acid sequences of pectin lyases of bacteria, fungi and oomycetes used in phylogenetic analyses**. Identical residues are marked with an asterisk (*). Dashes represent gaps introduced to preserve alignment. Conserved catalytic residues are indicated in boxes.

To elucidate the relationship of *Clpnl2 *from *C. lindemuthianum *with bacterial, oomycete and fungal pectin lyases, sequences reported in databases were analyzed. Protein or deduced amino acid PNL sequences from 14 fungal species including: basidiomycetes, ascomycetes and one oomycete species, three bacterial species, and a pectate lyase sequence from *A. thaliana *as an external group, were used to generate phylogenetic trees. Clustal alignment used for phylogenetic analysis (Figure 4) allowed to determine the location of amino acids expected to have a catalytic role in the PNLs [4,13]. Asp^154 ^and Arg^176 ^(numbered from *A. niger *PELA) are conserved in fungi and oomycetes, although Arg^176 ^could not be located in *P. griseoroseum *[GenBank: AF502280], and Arg ^236 ^is conserved in all analyzed sequences. Additionally, several conserved domains among the sequences of fungi and oomycetes were observed, and some of these were shared with bacterial amino acid sequences.

**Figure 5:**
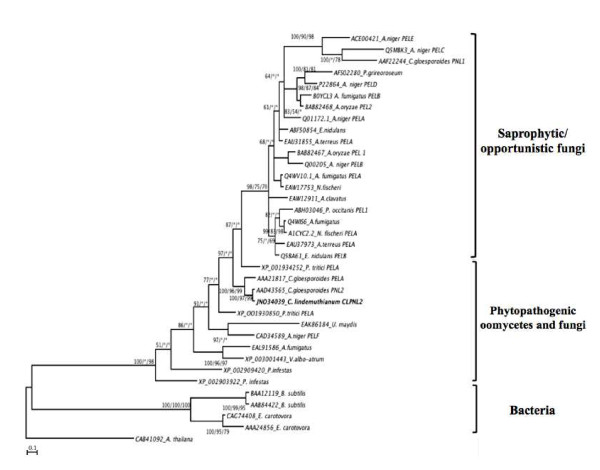
**Phylogenetic tree of pectin lyases**. The phylogeny shown is the Bayesian topology and branch lengths inferred using MrBayes vs. 3.1.2, with the Blosum 62 + G model. Numbers above the diagonal indicate posterior probability values from Bayesian analysis. Numbers below the diagonal indicate bootstrap percentage values from a bootstrap analysis inferred using the same alignment with PAUP*4.0 and Neighbor-J, respectively. *A. thaliana *pectate lyase was used as an outgroup. The asterisks represent branches that were not supported in 50% or more of the bootstraps. The scale bar represents the number of substitutions per site. The phylogenetic tree was edited using Dendroscope software [77].

The trees inferred by the maximum parsimony (MP) and neighbor-joining (NJ) methods showed less resolution than those built by Bayesian analysis, as they had a number of unresolved branches. The general topology obtained is represented by the Bayesian 50% majority rule consensus tree, in which the Bayesian posterior probabilities, MP and NJ bootstrap support are indicated on the branches (Figure 5).

Bayesian analysis allowed the separation of pectin lyases into two groups: one representing bacteria with 100% posterior probability and 100% bootstrap support for MP and NJ analysis, and the other one representing fungi and oomycetes with 100% posterior probability and 98% bootstrap support for NJ. In the group formed by bacteria, sequences from *Pectobacterium atrosepticum*, *P. carotovorum *and *Bacillus subtilis *cluster together with 100% posterior probability. This early separation between amino acid sequences of bacteria and those of oomycetes and fungi can be explained in terms of the evolution of lytic enzymes in these microorganisms for different purposes. Bacteria and some anaerobic fungi produce multi-enzymatic complexes called cellulosomes, which are anchored to the cell surface, allow the microorganisms to bind to lignocellulose substrates and increase the breakdown efficiency of cellulose, hemicellulose and pectin [62,63]. In contrast, in the majority of fungi and oomycetes, cellulases, pectinases and hemicellulases are not integrated in cellulosome complexes, and the pectin degradation is regulated by a multifunctional control system in which the enzymes act in a synergistic manner and are induced by monosaccharides or small oligosaccharides that are generated as products of the same enzymatic reactions [64,65].

The inferred tree also showed that the analyzed sequences of saprophytic/opportunistic fungi are clustered into a monophyletic group with 98% posterior probability and 75% and 70% bootstrap support for MP and NJ analyses, respectively. However, phytopathogenic fungi and oomycetes were not clustered together. This may be the result of a reduced representation of sequences in the analysis arising from the few PNL sequences reported for members of these groups.

*C. lindemuthianum *is found clustered with the amino acid sequences of PnlA and Pnl2 of the fungal pathogen *C. gloeosporioides *with 100% posterior probability for Bayesian analysis as well as 96% and 99% bootstrap support for MP and NJ analysis, respectively.

Pectin and pectate lyases fold into a parallel β-helix, in which a high structural conservation occurs in regions distant from the active site and particularly in those that contribute to the parallel β-helix architecture. The binding cleft and surroundings constitute the most divergent part of the molecule, which allows variation in substrate specificity [13,15]. On this background, the results of the phylogenetic analyses and the fact that the classification of the pectin lyases is based both on amino acid sequence similarities as well as their structural features [9], we believe that a structural comparison would help to strengthen the phylogenetic analysis and to establish a relationship between the genes encoding PNLs with their three-dimensional structures involved in carbohydrate binding.

### Multiple comparisons of protein structures

**Figure 6:**
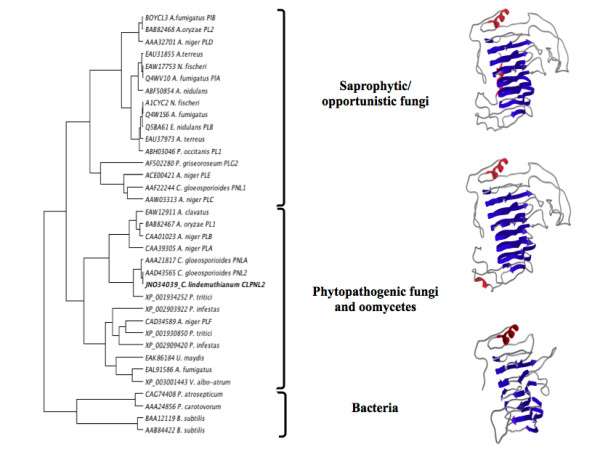
**Clustering the three-dimensional structures of pectin lyases**. The pectin lyase dataset was clustered by the un-weighted pair group method using the arithmetic mean (UPGMA) [53] with a similarity matrix obtained by the Voronoi contact method [51] using the ProCKSI-Server [52]. The tree image was generated using Dendroscope software [77]. A. Three-dimensional structure of PEL B from *A. niger *[PDB:1QCX]. B-C. Three-dimensional structures of the PNLs from *C. lindemuthianum *[GenBank: JN034039] and *P. carotovorum *[GenBank: AAA24856] respectively, predicted by homology modeling using the Swiss-Model Server [48].

Once the tertiary structure of *Clpnl2 *was predicted, the tertiary structures corresponding to the amino acid sequences used in phylogenetic analyses and covering the central body of the enzyme including the carbohydrate-binding site of these proteins were predicted and evaluated. The multiple comparisons of protein structures led to the formation of two clusters: one composed of the structures corresponding to the amino acid sequences of bacteria and another that was composed of fungal and oomycete structures (Figure 6). Furthermore, in agreement with the phylogenetic analyses, it was possible to distinguish the cluster formed mainly by sequences of fungi and oomycete pathogens, including Clpnl2, from the cluster formed by saprophytic/opportunistic fungi. Nevertheless, this analysis clustered the fungal sequences in two clearly defined groups: fungi and oomycete pathogens and saprophytic/opportunistic fungi. These results strongly support the notion that there is a close relationship between the tertiary structure of PNLs and the lifestyle of the microorganisms. The training of these groups was also observed for the elimination method FAST [66] and the hybrid heuristic URMS/RMS approach [67] using the ProCKSI-Server [52] (data not shown). Comparative modeling techniques and multiple comparisons of three-dimensional structures have been utilized for different purposes (e.g., searching for putative biological functions, drug design, protein-protein interaction studies). However, to our knowledge, this is the first study that uses a comparative analysis of protein structure in combination with a phylogenetic analysis to explore the evolution of lifestyle. We believe that a structural analysis can be an important tool for studying the evolution of microorganisms and their enzymes, since structural differences may reflect other important properties such as substrate specificity and others that can not be inferred from the analysis of amino acid sequences only.

### Expression analysis of *Clpnl2*

**Figure 7:**
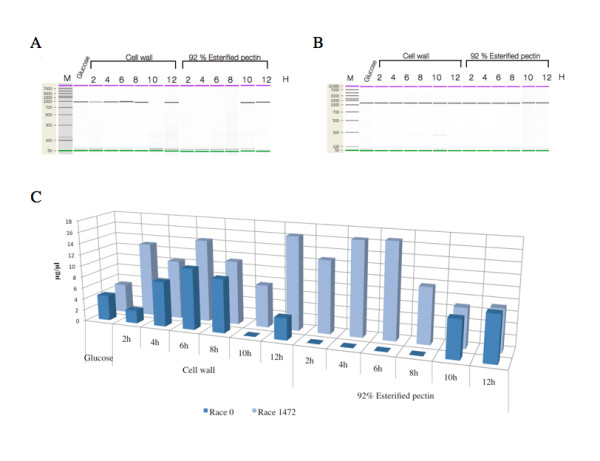
**Analysis of the relative gene expression of *Clpnl2 *in races 0 and 1472 of *C. lindemuthianum***. A-B. Gel-like images showing the expression of *Clpnl2 *in races 0 and 1472, respectively, on the different carbon sources tested. C. Semi-quantitative data for the expression of *Clpnl2 *in both races on the carbon sources. Total RNA was isolated from induced mycelia and amplified by RT-PCR with specific primers to yield the cDNA of *Clpnl2*. Amplification products were checked and quantified on a Bioanalyzer (2100 Agilent Bioanalyzer). The data were normalized using 18S rRNA as a control, and the results are expressed in μg/μl of amplified product.

Analysis of the *Clpnl2 *transcript in cells grown with glucose as the carbon source showed similar low basal levels of expression in the 0 and 1472 races (Figure 7C). When grown on cell walls, levels of *Clpnl2 *transcript in the pathogenic race, 1472, increased quickly after 2 h, reached a peak after 6 h, started to decrease and then again increased, giving a maximal value after 12 h of incubation (Figure 7B and 7C). Race 0 exhibited different expression kinetics: the amount of transcript peaked after 6 h and then fell to undetectable levels after 10 h (Figure 7A and 7C). At all time points between 2 and 8 h, expression levels were lower than those observed in the pathogenic race. The transcript was expressed again after 12 h but at levels that reached only 23% of those observed in the pathogenic race.

The differences between the two races were much more noticeable when 92% esterified pectin was used as the sole carbon source. Transcript expression in the pathogenic race started to increase rapidly, reached the highest levels after 4-6 h and then started to decline, giving a still significant increase at the end of the experimental period (Figure 7B and 7C). The maximum transcript levels on this substrate were clearly higher than those observed on glucose. In contrast, the levels of the *Clpnl2 *transcript in the non-pathogenic race remained undetectable after 8 h of incubation. Late expression occurred after 10-12 h of cultivation, reaching values comparable to those observed after 4 h of culture on cell walls (Figure 7A and 7C). These results agree with the differences found by Hernández et al. [34], who analyzed the extracellular activity of pectin lyase in both races of *C. lindemuthianum *under the same conditions employed in this study. When both races were grown with glucose, extracellular PNL activity was barely detected after 8 (race 1472) and 10 (race 0) days of incubation, as observed in this study. Plant cell walls from *P. vulgaris *induced a similarly low PNL activity in the two isolates after 7-8 days of incubation. When pectin esterified to 92% was used as the carbon source, the activity in the pathogenic race nearly doubled compared with the activity in the non-pathogenic race. Early transcription of genes encoding lytic enzymes and late detection of the corresponding activities is a well documented phenomenon in different fungi [8,30,65,68]. Apart from the presence of a regulatory system controlling gene expression, the production of active pectinase and probably other lyticases can be modulated by other mechanisms such as postranslational modification and protein transport [69]. These alternatives may help to explain the differences observed in this study.

The pectin lyase of the pathogenic race of *C. lindemuthianum *is able to degrade highly esterified pectin (92%), unlike that of the non-pathogenic race. Apparently, the differences between the pathogenic and non-pathogenic races of *C. lindemuthianum *occur as much at the expression level as at the level of enzymatic activity, and it is clear that the non-pathogenic and pathogenic races of *C. lindemuthianum *respond of different form to the carbon sources (except for glucose, where the mRNA of *Clpnl2 *and the active enzyme is synthesized at basal levels).

It has been proposed that the basal level of enzymatic activity breaks down the substrate, generating degradation products that further induce enzymatic activity [64]. A similar behavior has been observed in our laboratory for other enzymes that degrade cell walls, such as cellulases and the xylanase and β-xylosidase of *C. lindemuthianum *(unpublished data).

Several studies have reported that the pectinolytic enzymes play an important role in pathogenesis [70,71]. These are the first enzymes that act during the infection of the plant, causing extensive degradation of the cell wall and the main symptoms of the disease [72]. However, in addition to enzyme production, the sequence in which the enzymes are produced, the speed of synthesis, concentration and diffusion of enzyme are also fundamental aspects of the pathogenesis process [72]. The non-pathogenic race of *C. lindemuthianum *used in this work is unable to infect *P. vulgaris*, and thus its lifestyle is closer to that of a saprophytic fungus. Therefore, it is possible that the differences found between the non-pathogenic (0) and pathogenic (1472) races of *C. lindemuthianum *are related to the speed of activation of the lytic enzyme genes during the interaction with the host.

The number of pectin lyase sequences corresponding to different species of saprophytic/opportunistic fungi used in our analysis surpassed those of pathogenic oomycetes and fungi. This may be because more species of saprophytic/opportunistic have been studied and their degradation systems are better known. Alternatively, the enzymatic diversity may be the evolutionary effect of the heterogeneity of substrates that were encountered during interactions with an extended variety of hosts. For pectate lyases, it has been proposed that differences in the degree of pectin methylation can explain the existence of isozymes [4].

Pathogenic fungi and those who have close relationships with their host have developmental strategies that allow them to avoid the plant defenses and penetrate cell walls through the use of lytic enzymes. Plants also rely on strategies that allow them to detect and to defend against the attack of pathogens by producing inhibitors of these enzymes [70,73,74]. It is therefore possible that the evolution of unique enzymes was induced in pathogenic fungi and that a greater variability of these enzymes was induced in those fungi with a saprophytic lifestyle, which would explain the presence of amino acid sequences and tertiary structures corresponding to enzymes of saprophytic/opportunistic fungi located between the sequences of pathogenic fungi and oomycetes in the phylogenetic analysis and comparison of structures.

There is evidence that supports a relationship between lytic enzyme production and the lifestyles of fungi and oomycetes. For instance, the genome of the oomycete *Hyaloperonospora arabidopsidis *has lost several of its hydrolytic enzymes compared with *Phytophthora *sp., which is likely its ancestor [75,76]. According to an analysis of the hydrolytic profiles of saprophytic/opportunistic and pathogenic fungi using diverse substrates, the species of phytopathogenic fungi are more active than the non-pathogenic fungi on six of eight tested substrates [74]. It has also been observed that pathogenic fungi of monocotyledonous plants are better adapted to degrade the cell walls of monocotyledonous plants, and pathogens of dicotyledonous plants are better able to degrade the cell walls of dicotyledonous plants, reflecting the host preference [74].

## Conclusions

The *Clpnl2 *gene, which was cloned from a genomic library of *C. lindemuthianum*, is a unique copy and contains the characteristic elements of a pectin lyase of Family 1 of polysaccharide lyases.

Phylogenetic analyses showed an early separation between the enzymes of bacteria and those of fungi and oomycetes as well as a tendency of the amino acid sequences of fungi and oomycetes to cluster together according to their lifestyle. These results were confirmed by multiple comparison analysis of structures. According to these results, we believe that is possible that the diversity and nature of the substrates processed by these microorganisms play a determining role in the evolution of their lifestyle. In addition, our results showed that both races of *C. lindemuthianum *express the *Clpnl2 *gene, although some differences are observed in the timing and level of expression: the pathogenic race responds faster and at higher levels than the non-pathogenic race. This suggests that there are at least two levels of determination of the lifestyle of the microorganisms: one related to the evolution of the enzymes and one concerning the regulation of the expression of the enzymes. In our model, one race of *C. lindemuthianum *behaves as a hemibiotrophic pathogen and, according to its inability to infect bean, the other race behaves as a saprophyte. Although this study included the analysis of pectin lyase 2 only, we have observed this behavior with other enzymes of the complex involved in the degradation of the cell wall suggesting that it may be a general phenomenon. The differences at this level can be part of the general response of the fungi to host components. However future studies comparing the enzymatic complex of degradation of more fungi species with different lifestyles are needed to confirm this hypothesis.

Finally, we consider this type of information to be of great importance for the study of the biotechnological potential of these enzymes, as the efficiency of the enzymes could depend on the complexity of the vegetal material to be processed and the lifestyle of organism that is the source of enzymes and/or genes.

## Authors' contributions

ALM, MGZP and UCS carried out the experiments. ALM and NCC carried out data analysis. ALM, MGZP and HCC conceived and designed the study, guided data analysis, interpretation, and discussion, and wrote the manuscript with comments from ELR and RLG. ELR participate in biochemical interpretation of data and RLG participate in genomic library construction. All authors read and approved the final manuscript.

## Acknowledgements

The authors thank the financial support provided by the FOMIX CONACYT-Gobierno del Estado de Michoacán (project 2009-05 Clave 116208 to HCC) and CONACYT (scholarship granted to ALM and UCS). We thank Gerardo Vázquez Marrufo by its comments to manuscript.
